# Maternal Vaccination in Lithuania: A Cross-Sectional Study

**DOI:** 10.3390/vaccines14040363

**Published:** 2026-04-18

**Authors:** Gabija Matuzaitė, Diana Ramašauskaitė

**Affiliations:** 1 Faculty of Medicine, Vilnius University, 01513 Vilnius, Lithuania; 2 Institute of Clinical Medicine, Clinic of Obstetrics and Gynecology, Faculty of Medicine, Vilnius University, 01513 Vilnius, Lithuania

**Keywords:** influenza, pertussis, respiratory syncytial virus, maternal vaccination, Lithuania

## Abstract

*Objective:* Influenza and pertussis vaccines are recommended during pregnancy; however, uptake remains insufficient in many European countries, increasing the risk of preventable infections. Recent recommendations for maternal respiratory syncytial virus vaccination have been endorsed by scientific societies. This study evaluated maternal vaccination coverage, knowledge, attitudes, and factors influencing vaccine uptake among Lithuanian women. *Methods:* A retrospective cross-sectional online survey was conducted between 4 and 14 November 2025 in Lithuania among women aged 18–55 years with at least one previous pregnancy. The questionnaire contained 29 questions on sociodemographic characteristics, obstetric history, vaccination history, attitudes, and informational sources influencing decisions. Internal reliability was confirmed (Cronbach’s α = 0.83). Descriptive statistics were used to summarize the data. Associations between categorical variables were assessed using the Chi-square test or exact tests (Fisher’s exact or Fisher–Freeman–Halton). Binary and multivariable logistic regression analyses were performed to evaluate factors associated with self-reported vaccination uptake and the relationship between influenza and pertussis vaccination. Odds ratios with 95% confidence intervals were calculated. Statistical significance was set at *p* < 0.05. *Results*: A total of 241 women participated. Self-reported vaccination coverage during pregnancy was 28.7% for influenza, 43.8% for tetanus–diphtheria–pertussis, and 4.2% for respiratory syncytial virus. Physician’s recommendation was the strongest predictor: women advised to vaccinate were 17.0 times more likely to receive influenza, 16.5 times more likely to receive pertussis, while RSV vaccination occurred almost exclusively among women who reported receiving a physician’s recommendation. Higher uptake was associated with younger maternal age and university education. Reasons for declining vaccination were avoidance of medical interventions and concerns about safety or side effects. *Conclusions*: Maternal vaccination coverage in Lithuania remains low despite public funding and national recommendations. Strengthening provider communication, improving information strategies, and integrating vaccination counseling into routine antenatal care may increase uptake and enhance maternal and neonatal protection.

## 1. Introduction

### 1.1. Importance of Maternal Vaccination

During pregnancy, the maternal immune system undergoes a process of immune modulation to protect both the mother and the developing fetus, thereby supporting a successful and uncomplicated pregnancy [1,2]. During early pregnancy, there is a physiological shift toward a Th2-dominant profile [1,3]. This immunological shift increases maternal vulnerability and raises concern for potential pathogen exposure to the fetus and newborn, creating a need for strategies that can provide immune protection during this critical period [4].

Prenatally and in the early neonatal period, the newborn is exposed to a wide variety of previously unencountered pathogens, many of which can cause severe disease in the absence of immune protection [5,6]. According to the European Center for Disease Prevention and Control (ECDC), World Health Organization (WHO) pregnant and postpartum women are at increased risk of severe complications from seasonal and pandemic influenza, as well as from SARS-CoV-2 infection [7]; in neonates, other major infectious causes of hospitalization include life-threatening pertussis and respiratory syncytial virus (RSV) infections [8,9].

The infant’s own immune system is immature and unable to mount robust responses to many infections. Protection during this vulnerable period relies primarily on maternal antibodies transferred both transplacentally during pregnancy (mainly IgG) and postnatally through breast milk (predominantly IgA) [1,10]. Several vaccines are administered only after the first few months of life, including vaccines against diphtheria, tetanus, pertussis, rotavirus, Haemophilus influenza type b, pneumococcus, and poliovirus, thereby leaving a window of susceptibility in early infancy [11]. Vaccination during pregnancy is associated with reduced risks of adverse maternal and perinatal outcomes—including preterm birth, pneumonia, hospitalization, intensive care admission, stillbirth, small-for-gestational-age infants, and maternal or fetal death—and provides passive protection to the infant via transplacental antibody transfer until the infant’s own vaccinations take effect [1,12,13,14].

### 1.2. Vaccines During Pregnancy

In 2012, the ECDC issued a public health guidance to vaccinate pregnant women from seasonal influenza [15]. The ECDC vaccine scheduler presents country-specific national recommendations for maternal pertussis (Tdap) vaccination across European Union and European Economic Area (EU/EEA) countries [16]. No influenza vaccine is licensed for infants less than 6 months of age, and the first pertussis vaccine is licensed to be given at 2 months of age; hence, vaccination during pregnancy is a key strategy to protect both mother and infant [14]. Maternal vaccination against RSV, a recently introduced strategy, is gaining increasing attention and has already been incorporated into national recommendations in several EU/EEA countries. Passive immunization of the infant with long-acting monoclonal antibodies may be used for protection against RSV infection. According to the ECDC, only one preventive strategy should generally be selected [17]. Live-attenuated vaccines, such as those against measles, mumps, and rubella (MMR), are advised prior to conception. These national recommendations are aligned with guidance from the WHO [18,19].

### 1.3. Burden of Vaccine-Preventable Diseases

Influenza, particularly in infants under 6 months of age, is associated with the highest hospitalization rates among pediatric age groups [20]. Among hospitalized influenza-positive pediatric patients, secondary bacterial infections—including primary or secondary pneumonia—occur in 28–36% of cases, cardiac complications such as myocarditis and pericarditis have also been reported. Neurologic manifestations, including seizures and encephalopathy, occur in approximately 7–10% of hospitalized cases, with febrile seizures (5%), encephalopathy (1.7%), and nonfebrile seizures (1.2%) representing the most frequently reported neurological complications [21,22].

The number of reported pertussis cases increased from 25,000 in 2023 to more than 32,000 in the first quarter of 2024, with the majority of cases and fatalities occurring in infants [23]. This underscores the critical importance of maternal immunization for neonatal health, as *Bordetella pertussis* infection causes severe, paroxysmal coughing (“whooping cough”) and is particularly dangerous for infants under six months of age, potentially leading to apnea, hypoxia-induced seizures, pulmonary hypertension, pneumonia, otitis media, and death [24,25].

Approximately 2–3% of infants younger than 6 months are hospitalized for RSV each year [26,27]. Severe RSV-associated lower respiratory tract illness (LRTI), such as bronchiolitis or pneumonia, peaks at 2–3 months of age and is a leading cause of infant mortality within the first 6 months of life. As no specific antiviral therapy is routinely available for RSV, management remains largely supportive, including supplemental oxygen and respiratory support when needed [28].

### 1.4. Maternal Vaccination Recommendations in Lithuania

In Lithuania, national recommendations from the Ministry of Health include two maternal vaccines: seasonal influenza and pertussis. Influenza vaccination for pregnant women has been government-funded since the 2011–2012 influenza season; pertussis vaccination has been publicly funded since 2023. Although the RSV vaccine is not yet nationally recommended or government-funded, the Lithuanian Society of Obstetricians and Gynecologists (LSOG) recommends its administration at least 4 weeks before the expected date of delivery [29]. Some European countries, including Lithuania, have implemented publicly funded RSV monoclonal antibody programs, currently restricted to infants at increased risk of severe disease rather than applied universally [17]. Table A1 in Appendix A represents vaccines that are recommended for pregnant women in Lithuania [30,31].

### 1.5. Maternal Vaccination Uptake in Lithuania

Despite public funding, influenza vaccine uptake among pregnant women remains critically low. The data from the Lithuanian Institute of Hygiene indicate that in 2023, only 7.8% of pregnant women received the influenza vaccine and 4.8% received the Tdap vaccine during pregnancy [32].

The ECDC report on national vaccination coverage across the EU/EEA for the 2024/2025 season indicated that influenza vaccine uptake among pregnant women in Lithuania was 9.9%, compared with an average of 22% across reporting European countries [33].

Data on RSV vaccine uptake in Lithuania is currently unavailable.

### 1.6. Challenges—Vaccine Hesitancy

Vaccine hesitancy, often driven by misinformation and disinformation not supported by scientific evidence, has contributed to persistent public skepticism regarding maternal immunization. A 2016 survey of obstetricians and gynecologists reported that the most common reasons pregnant women declined vaccination were the belief that influenza vaccination causes illness (48%), the perception of low personal risk for vaccine-preventable diseases (38%), general concerns about vaccines (32%), a preference for a “natural” pregnancy (31%), and fear that vaccination during pregnancy could cause autism in the child (25%) [34]. Healthcare providers’ and policymakers’ need to understand where hesitancy or other barriers occur along the continuum of pregnancy care and to design effective interventions.

The aim of this study was to assess self-reported uptake of maternal influenza, Tdap, and RSV vaccinations among women in Lithuania, and to identify factors associated with vaccination uptake, including sociodemographic characteristics and physician recommendation, as well as to evaluate the relationship between uptake of different maternal vaccines.

## 2. Materials and Methods

### 2.1. Study Design and Setting

A retrospective cross-sectional survey was conducted between 4 and 14 November 2025. No validated maternal vaccination data-collection form was available; therefore, a unique Lithuanian-language questionnaire was developed. The self-administered questionnaire was anonymous and included an informed consent statement. The survey was distributed online using nationwide convenience sampling. The questionnaire link (Google Forms, Google LLC, Mountain View, CA, USA) was shared via Facebook groups and online forums related to pregnancy, expectant mothers, and child health. Based on publicly available membership data of the groups and online forums, the estimated number of potentially targeted individuals was 209,103. Groups explicitly focused on pro-vaccination or anti-vaccination advocacy were intentionally avoided to reduce ideological sampling bias. A detailed description of the group selection, including keyword identification, inclusion and exclusion criteria, and assessment of group neutrality, is provided in the Supplementary Materials (Figure S1). Recruitment was nationwide, and no geographic or urban–rural stratification was applied. No incentives were offered for respondents.

### 2.2. Survey Development and Administration

The final questionnaire comprised 29 questions covering sociodemographic characteristics, obstetric and vaccination histories, attitudes toward vaccines, and sources of vaccine information. A pilot test was performed with 10 participants; no changes were made. Reliability was assessed using Cronbach’s alpha (0.83), applied only to items assessing attitudes and beliefs for which internal consistency is appropriate. The required sample size was calculated using G*Power version 3.1 (Heinrich Heine University, Düsseldorf, Germany) to be 384 respondents, with a 95% confidence level and a 5% margin of error. A total of 241 responses were collected, corresponding to an actual margin of error of approximately 6.3%, indicating slightly reduced precision. The questionnaire included both mandatory and optional items, with conditional display based on participants’ previous responses. Optional or non-applicable questions could be skipped, resulting in different sample sizes across analyses.

The questionnaire consisted of 29 items grouped into four domains: sociodemographic characteristics, obstetric and pregnancy history, vaccination status and healthcare provider recommendations during pregnancy (influenza, Tdap and RSV), and attitudes toward vaccination and sources of information. Physician recommendation was assessed by self-report. Vaccination status questions were primarily structured as closed-ended yes/no items with an additional do not remember option where applicable, while attitudes and beliefs were assessed using multiple-choice and Likert-type response formats. Education level was categorized according to the Lithuanian education system. University degree refers to university-based academic education leading to a bachelor’s or master’s degree, whereas higher education institutions refer to non-university tertiary education obtained at colleges, which provide professionally oriented short-cycle higher education. The complete questionnaire is available in the Supplementary Materials section.

Several items were adapted from the 2023 National Public Health Center questionnaire on Lithuanian residents’ knowledge and opinions about vaccination, conducted by the Infectious Disease Control Division of the Vilnius Department of the National Public Health Center under the Ministry of Health. Permission to use these items was obtained from the Quality Management and Communication Department of the National Public Health Center. Questions corresponding to items 19 and 23 were linguistically adapted to specifically address pregnant women rather than the general population. Questions 22 and 24–29 were used in their original form without modification.

The primary outcome variables were self-reported vaccination uptake for influenza, Tdap and RSV. Secondary outcome variables included attitudes toward vaccination, perceived barriers, and sources of vaccine information. Statistical analyses were conducted using SPSS version 31.0.0.0 (IBM Corp., Armonk, NY, USA), Microsoft Excel (Microsoft Corp., Redmond, WA, USA), and GraphPad Prism version 10.6.1 (GraphPad Software, Boston, MA, USA). Associations between categorical variables were assessed using the Chi-square test of independence when all expected cell counts were ≥5. Fisher’s exact test was applied for 2 × 2 contingency tables when at least one expected cell count was <5. For contingency tables larger than 2 × 2 with sparse data or expected cell counts <5, the Fisher–Freeman–Halton exact test was used. These criteria ensured appropriate handling of small sample sizes and low-frequency categories. Multivariable logistic regression analysis was performed to examine the association between reported influenza and Tdap vaccination, adjusting for maternal age, education level, and physician recommendation. Logistic regression analysis was performed to assess the association between influenza and Tdap vaccination. *p*-value < 0.05 was considered statistically significant. Associations were expressed as odds ratios (OR) with 95% confidence intervals (CI).

### 2.3. Study Groups and Eligibility Criteria

The inclusion criteria were women aged 18–55 years who had experienced at least one pregnancy (past or current). The upper age limit of 55 years was chosen to encompass the general reproductive age range, as this approximates the median age of menopause in the population. Participants reported vaccination behaviors related to any previous pregnancy (past or current). The questionnaire did not restrict responses to the most recent pregnancy or to a predefined time frame. For the purposes of this study, vaccination during pregnancy was defined as receipt of at least one dose of the respective vaccine at any time during pregnancy, regardless of trimester.

Tdap vaccination became publicly funded and formally recommended for pregnant women in Lithuania starting in 2023. Accordingly, analyses related to Tdap vaccination were restricted to participants whose pregnancies began in 2023 or later. Eligibility for this subgroup was determined based on self-reported pregnancy timing in the questionnaire. As a result, the analytic sample for Tdap-related analyses comprised 176 participants, whereas analyses for influenza and RSV vaccination included the full study sample (*n* = 241).

**Figure 1:**
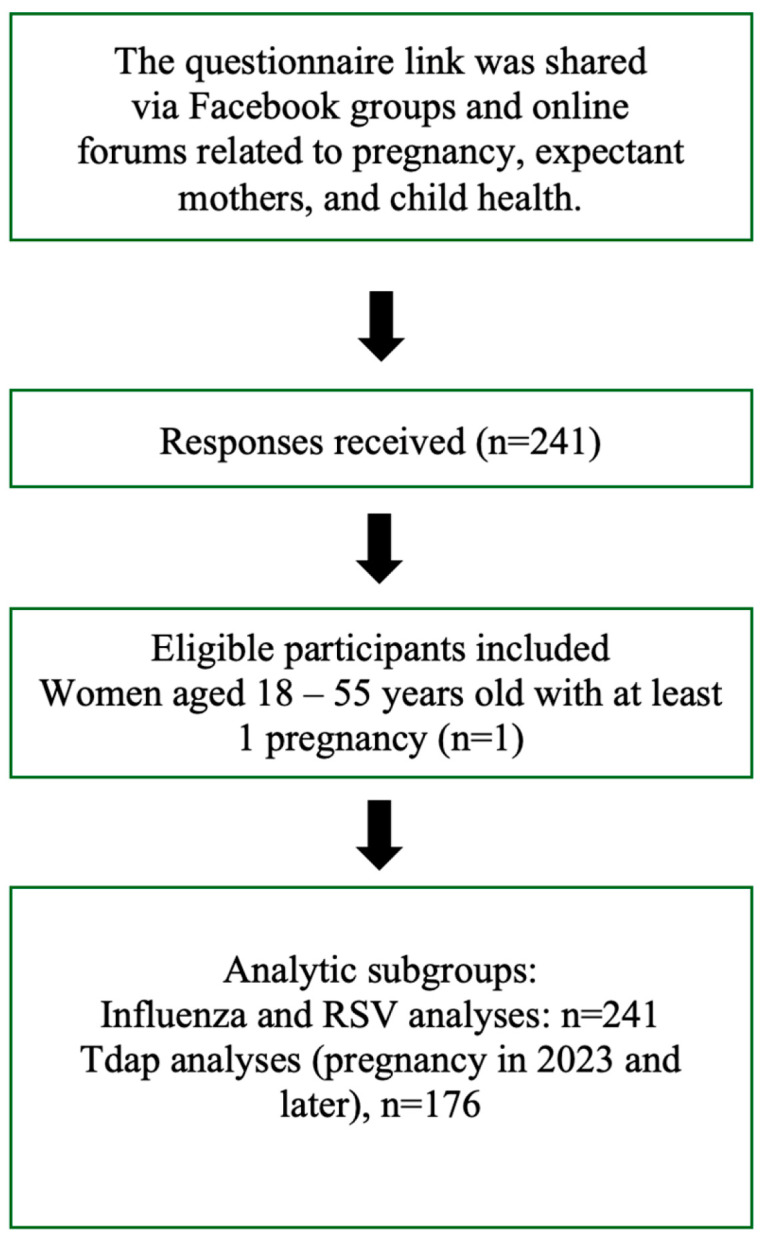
Flowchart of participant recruitment and analytic subgroups.

In this study, participants were not assigned to vaccination groups by study design. Vaccination status was determined based on self-reported vaccination history related to pregnancy. Participant recruitment, inclusion, and derivation of analytic subgroups are illustrated in Figure 1.

## 3. Results

### 3.1. Sociodemographic, Pregnancies, Miscarriages, and Birth Rates

A total of 241 respondents completed the questionnaire. The majority of participants reported having a university degree (81.7%), followed by graduates of higher education institutions (8.7%), a high school education (8.7%), and a small proportion with compulsory education up to grade 10 (0.8%). The baseline characteristics of the study population, including age distribution, number of pregnancies, miscarriages, and live births, are summarized in Appendix A (Table A2).

### 3.2. Primary Outcomes

The primary outcome variables included self-reported vaccination rates for influenza, Tdap and RSV. The influenza vaccine was administered to 28.7%, and 41.1% of all respondents reported receiving vaccination recommendations. The data were not stratified by season. The Tdap vaccine was administered to 43.8% of women, with recommendations being reported up to 51.1%. The RSV vaccine was administered to 4.2% of women, and 6.2% reported receiving recommendations for this vaccination. Percentages for influenza and RSV vaccination were calculated from the total number of respondents. Tdap vaccination percentages were calculated from the group of women (*n* = 176) who answered the pertussis vaccination question, which applied only to pregnancies beginning in 2023 or later. The results are summarized in Table 1. Binary logistic regression analysis demonstrated a strong association between influenza and Tdap vaccination. Women who reported influenza vaccination had significantly higher odds of reporting Tdap vaccination (OR = 11, 95% CI: 5.14–23.42, *p* < 0.001).

#### 3.2.1. Correlation Between Recommendations and Vaccination Rates

The questionnaire findings indicate that less than half of the respondents received a doctor’s recommendation for vaccinations: influenza (41.1%), Tdap (51.1%), and RSV (6.2%). To further evaluate whether receiving a medical recommendation influences actual vaccination behavior, statistical analyses were conducted to assess the association between physician recommendations and vaccine uptake. In Lithuania, antenatal care is primarily provided by obstetrician–gynecologists, while family physicians usually consult pregnant women only once at the beginning of pregnancy and only if specific indications arise. The data presented in Table 1 were used for this analysis.

Unadjusted statistical analyses (contingency table-based) demonstrated a statistically significant association between physician recommendation, influenza and Tdap vaccination during pregnancy (*p* < 0.001, Chi-square test of independence). Women who reported receiving a physician recommendation were significantly more likely to be vaccinated during pregnancy, with approximately 19-fold higher odds of influenza vaccination (OR = 19.4, 95% CI: 8.3–45) and 13-fold higher odds of pertussis (Tdap) vaccination (OR = 12.7, 95% CI: 5.9–27.4) compared with those who did not receive such a recommendation. A significant association was also found between physician recommendation and RSV vaccination during pregnancy (*p* < 0.001, Fisher’s exact test). RSV vaccination occurred almost exclusively among women who reported receiving a physician recommendation, resulting in a large odds ratio with a wide confidence interval (OR = 110.9, 95% CI: 18.7–658.5), reflecting small subgroup sizes and limited precision.

#### 3.2.2. Education, Maternal Age, and Vaccination Rates Correlation

Statistical analysis was conducted to determine whether education level or maternal age was significantly associated with vaccination uptake during pregnancy. When comparing vaccination rates by education level, no statistically significant differences were observed for influenza (*p* = 0.340) or RSV vaccination (*p* = 0.330). A statistically significant association was found between education level and Tdap vaccination (*p* = 0.020), indicating that women with a university degree were more likely to receive the Tdap vaccine. Analyses by maternal age revealed significant associations for both influenza (*p* < 0.001) and Tdap vaccines (*p* = 0.010), with younger women being more likely to be vaccinated. For RSV vaccination, no statistically significant association with maternal age was found (*p* = 0.067). A summary of these findings is presented in Supplementary Material (Table S1).

#### 3.2.3. Multivariable Analysis of Factors Associated with Influenza and Tdap Vaccination

Multivariable logistic regression analysis was performed to determine whether the association between physician recommendation and vaccination uptake persists after adjustment for potential confounding factors, including maternal age and education, with influenza and Tdap vaccination uptake. Maternal age, education level, and physician recommendation were included as covariates. Adjusted estimates and results are presented in Table 2. For influenza vaccination, physician recommendation remained the strongest predictor (OR = 17, *p* < 0.001). Similarly, for Tdap vaccination, physician recommendation remained a strong independent predictor (OR = 16.5, *p* < 0.001), and education level demonstrated a significant overall association (*p* = 0.038).

Differences between unadjusted and adjusted estimates suggest the influence of potential confounding; however, physician recommendation remained a strong independent predictor of vaccination uptake after adjustment.

### 3.3. Secondary Outcomes

#### 3.3.1. Respondents’ Reasons for Not Vaccinating During Pregnancy

To further assess the factors influencing vaccine hesitancy, respondents who reported refusing the recommended vaccines during pregnancy (influenza, Tdap, or RSV) were asked to indicate their reasons for not vaccinating. Questionnaire options were provided to capture both safety-related concerns and personal beliefs. The most frequently reported reason was the general avoidance of medications or vaccines (21.1%). Other common reasons included the perception that there is insufficient data on vaccine safety (19.3%), concerns about vaccine-related side effects (15%), and fear of complications resulting from vaccination (14.1%). A smaller proportion of respondents believed that children should acquire immunity through natural infection (8%). Additional reasons for not vaccinating are presented in Supplementary Material (Table S2).

#### 3.3.2. Information Sources Influencing Vaccination Decisions During Pregnancy

The questionnaire aimed to identify which sources most strongly influenced respondents’ vaccination decisions during pregnancy. Figures S2 and S3 in Supplementary Material illustrate the distribution of the most frequently reported sources used in deciding whether to vaccinate. For the decision to vaccinate, the majority of women based their choice on official sources, such as the Lithuanian Ministry of Health and the National Public Health Center (32.9%). The second most trusted source was advice from a family physician (27.2%), followed by scientific articles and literature (19.1%). For the decision not to vaccinate, women most often gathered information from official sources (20.9%), a family physician (19.2%), and the scientific literature (15.3%) as their primary sources guiding their decision Overall, the findings indicate that both vaccination and non-vaccination decisions were primarily influenced by formal and professional information sources, social media and peer influence played only a minor role in decision-making.

To evaluate respondents’ general knowledge and perceptions of vaccination, several questions assessed their personal beliefs, attitudes, and misconceptions regarding immunization. The respondents demonstrated generally positive attitudes toward vaccination, although some uncertainty regarding vaccine safety and mechanisms persisted. Most participants agreed that vaccination is beneficial (67.7%) and vaccines are safe (58.9%). Detailed distributions of attitudes, beliefs, and knowledge are presented in Supplementary Material (Figure S4 and Tables S3–S7).

## 4. Discussion

This cross-sectional study assessed self-reported vaccination coverage among pregnant Lithuanian women and identified key factors influencing vaccine uptake. The results indicated low vaccination uptake for influenza, Tdap and RSV vaccines among the study sample. Physician recommendation was associated with higher vaccination uptake, particularly for influenza and Tdap. The magnitude of the observed OR for RSV vaccination should be interpreted with caution. The high estimate reflects the low awareness and uptake, with self-reported vaccination occurring predominantly among women who received a physician recommendation. The small number of vaccinated individuals contributes to the wide CI observed, indicating limited statistical precision. Higher vaccination rates were also observed among women with higher education and younger maternal age, likely reflecting greater health awareness and engagement in preventive care. Women who reported influenza vaccination during pregnancy had higher odds of receiving Tdap vaccination, indicating that acceptance of one maternal vaccine may be associated with increased uptake of others. The main reasons for vaccine refusal were the general avoidance of medical interventions, when possible, concerns about insufficient safety data, and potential side effects or complications. Although most respondents cited official health sources (the Lithuanian Ministry of Health or the National Public Health Center) as their basis for decision-making, these institutions do not publish any recommendations discouraging vaccination. This suggests that some women may misinterpret or attribute false information to authoritative sources, underscoring the need for clear, consistent, and evidence-based communication about maternal vaccination.

The primary reasons for vaccine refusal reported in other studies closely align with our findings. A narrative review by Stephanie L. Mitchell et al. identified fear of side effects or adverse events and lack of confidence in vaccine safety as the most frequent reasons for hesitancy among pregnant women [35]. The María Isabel Fernández–Cano et al. study found that the main reasons for not receiving the influenza vaccine were the belief that the vaccine is ineffective and concerns about its safety for the fetus [36]. Additional factors contributing to non-vaccination include low perceived risk of infection during pregnancy, lack of prior vaccination experience, vaccine cost, and, more recently, spousal hesitancy [35]. In our study, cost accounted for only 0.3% of reported reasons for refusal. In Lithuania, influenza and Tdap vaccines are publicly funded, whereas the RSV vaccine is not, which may further discourage its uptake.

Vaccine hesitancy, reflected in the discrepancy between recommended vaccines and their actual uptake, can be reduced through several key strategies: consistent physician recommendation, dissemination of information from reliable sources, and strengthening maternal education and health literacy. A cross-sectional study conducted in Turkey reported that receiving a physician’s recommendation was significantly associated with lower vaccine-hesitancy scores during pregnancy [37]. The results match our findings, showing that 85.6% of Tdap vaccinations occurred following a doctor’s recommendation, compared with 73% of Turkish respondents who stated they would accept tetanus vaccination if advised by their physician. A higher proportion of pregnant women who were initially hesitant about influenza vaccination ultimately chose to be vaccinated after receiving a direct provider recommendation or referral [38]. The most common reason for not receiving pertussis vaccination was lack of information or unawareness of vaccination recommendations (24%), whereas influenza vaccination was most frequently declined due to perceived low efficacy (28%) and concerns about side effects (22–23%) [39]. In this study, 5.2% of respondents reported declining vaccination due to perceived lack of vaccine efficacy. Although this proportion is lower than that reported in previous studies, both findings indicate that doubts about vaccine effectiveness remain a recurrent barrier to maternal immunization. These findings highlight that proactive physician engagement and clear, evidence-based communication are essential to address misinformation and improve vaccine confidence during pregnancy.

Education level and reliance on official information sources were associated with higher health literacy scores. In our study, Tdap vaccination was associated with education—women with a university degree were more likely to be vaccinated. Previous research demonstrates that higher health literacy is more common among married women, those with higher education, urban residents, and those with better socioeconomic status [37]. Other studies highlight the importance of maternal risk conditions and comorbidities in vaccination decisions, reporting that asthma was the variable most strongly associated with maternal pertussis (Tdap) vaccination [36]. Sociodemographic and clinical characteristics can influence maternal vaccination behavior and should be considered in future public-health interventions.

A total of 28.7% of pregnant women reported receiving the influenza vaccine, 43.8% received Tdap, and 4.2% received the RSV vaccine in Lithuania. This demonstrates a relatively low level of vaccine uptake when compared with reports from other European countries. According to the 2024–2025 ECDC report, 30 EU/EEA countries have national recommendations for influenza vaccination during pregnancy. Despite these recommendations, vaccination coverage among pregnant women varies across countries, ranging from less than 2% in Hungary to over 60% in Spain. Coverage has been reported at 58.9% in Finland, 35% in Ireland, and 20–23% in Denmark, Portugal, and the Netherlands [33]. Tdap vaccination coverage among pregnant women varies across Europe, from as low as 1.6% in the Czech Republic and 6.5% in Slovenia to much higher levels such as 88.5% in Spain, 69% in Denmark, and 64.3% in Belgium [40]. Across most European countries, Tdap uptake exceeds influenza vaccination. This raises important questions about the reasons behind this difference—whether pregnant women view pertussis as a greater risk than influenza, or whether factors such as national policies and healthcare provider recommendations play a larger role in contributing to this disparity. The low RSV vaccination rate observed (4.2%) was expected, given that the maternal RSV vaccine was approved in the European Union in 2023 and has not yet been incorporated into Lithuania’s national immunization program. Maternal vaccination coverage in Lithuania remains among the lowest in Europe, mirroring trends in several Central and Eastern European countries.

This study offers one of the earliest detailed evaluations of self-reported maternal vaccination coverage in Lithuania, including influenza, Tdap and RSV. The study uses a structured and pilot-tested questionnaire with good internal reliability (Cronbach’s α = 0.83), allowing evaluation of vaccine uptake and the underlying reasons for acceptance or refusal. Several limitations should be acknowledged. The questionnaire did not collect the specific year of pregnancy or account for seasonality, limiting the ability to assess whether vaccines were recommended during the respondents’ pregnancies. This may have influenced the interpretation of vaccination coverage across all respondents. This limitation does not affect the accuracy of the Tdap vaccination analysis, as only respondents whose pregnancies started in 2023 or later were included in that part of the analysis, corresponding to the year when Tdap vaccination was officially introduced and publicly funded for pregnant women in Lithuania. The sample size (*n* = 241) did not reach the target, which may reduce precision for subgroup analyses. The participation rate was low (241 out of 209,103), which may limit the representativeness of the sample and restrict the applicability of the findings. The study relied on self-reported data and convenience sampling without a predefined sampling frame, recall, reporting, and selection bias may be present, which should be considered when interpreting the findings. Despite these limitations, the findings provide a useful national baseline for understanding maternal vaccination attitudes in Lithuania and highlight modifiable factors—particularly healthcare-provider recommendations and public education.

## 5. Conclusions

This study provides information on self-reported maternal vaccination coverage in Lithuania in 2025. The influenza vaccination was reported by 28.7% of respondents, pertussis by 43.8%, and RSV by 4.2%. A physician’s recommendation is the most important factor influencing vaccination uptake. Higher vaccination rates were observed among younger women with a university education, indicating an association between education level and vaccine uptake. The main reasons for refusal were avoidance of medical interventions and concerns about vaccine safety or potential side effects.

A significant association between influenza and Tdap vaccination suggests that acceptance of one maternal vaccine may increase the likelihood of uptake of others.

Maternal vaccination rates in Lithuania remain low, which is consistent with trends in other Central and Eastern European countries. Strengthening healthcare-provider engagement, education of medical staff, improving the clarity of official communication, and integrating vaccination counseling into routine antenatal care could substantially increase vaccine uptake and enhance maternal and neonatal protection.

## Figures and Tables

**Table 1:** Recommendations and vaccination rates.

Vaccine	Recommended by Doctor (n)	Vaccinated During Pregnancy (n)	*p*-Value	OR (95% CI) *
Influenza (*n* = 241)	Yes: 99	Yes: 69	<0.001	19.4 (8.3–45.0)
	No: 128	No: 171		
	Does not remember: 14	Does not remember: 1		
Tdap (*n* = 176)	Yes: 90	Yes: 77	<0.001	12.7 (5.9–27.4)
	No: 80	No: 96		
	Does not remember: 6	Does not remember: 3		
RSV (*n* = 241)	Yes: 15	Yes: 10	<0.001	110.9 (18.7–658.5)
	No: 198	No: 219		
	Does not remember: 28	Does not remember: 12		

* Participants who selected “does not remember” were excluded from the analysis. OR represents the odds of vaccination among women who received a physician recommendation compared to those who did not.

**Table 2:** Multivariable logistic regression analysis.

	Influenza Vaccination	Tdap Vaccination
Physician recommendation	*p* < 0.001 (OR = 17, 95% CI: 7.34–39.13)	*p* < 0.001 (OR = 16.5, 95% CI: 7.14–38.00)
Maternal age	*p* = 0.220	*p* = 0.686
Education level	*p* = 0.443	*p* = 0.038

## Data Availability

The research data are available from the first author upon reasonable request.

## Supplementary Materials

The following supporting information can be downloaded at: https://www.mdpi.com/article/10.3390/vaccines14040363/s1, Figure S1: Selection of online groups for survey distribution; Figure S2: Information sources influencing the decision to vaccinate during pregnancy; Figure S3: Information sources influencing the decision not to vaccinate during pregnancy; Figure S4: “Is it better to acquire immunity through natural infection or vaccination?’’ Responses according to respondents; Table S1: Education, maternal age and vaccination rates; Table S2: Reasons for not vaccinating; Table S3: “Should the vaccination for pregnant women be mandatory?” Responses according to respondents; Table S4: Understanding the knowledge respondents of vaccine mechanisms; Table S5: “Vaccinations are beneficial because they protect against infectious diseases”. Responses according to respondents; Table S6: “Vaccines are safe”. Responses according to respondents; Table S7: “Vaccines are only beneficial to pharmaceutical companies and not to vaccinated people”. Responses according to respondents.

## Abbreviations

The following abbreviations are used in this manuscript:

ACOG	American College of Obstetricians and Gynecologists
CI	Confidence Interval
COVID-19	Coronavirus Disease 2019
ECDC	European Center for Disease Prevention and Control
EU	European Union
G*Power	Statistical Power Analysis Program
IgA	Immunoglobulin A
IgG	Immunoglobulin G
LRTI	Lower Respiratory Tract Infection
LSOG	Lithuanian Society of Obstetricians and Gynecologists
MMR	Measles, Mumps, and Rubella
NIS	National Immunization Schedule
OR	Odds Ratio
RSV	Respiratory Syncytial Virus
RSVPreF3-Mat	Respiratory Syncytial Virus Prefusion F Protein-Based Maternal Vaccine
SAGE	Strategic Advisory Group of Experts on Immunization
SPSS	Statistical Package for the Social Sciences
Tdap	Tetanus, Diphtheria, and Acellular Pertussis
Th	T helper cell
WHO	World Health Organization

## Appendix A

**Table A1:** Vaccination recommendations for pregnant women in Lithuania.

	Influenza Vaccination	Tdap Vaccination	RSV
Lithuania	Before (ideally by the end of October) and during the flu season, not specific to trimester [30], during every pregnancy.	From the 28th–36th week during every pregnancy	No national recommendations [30].

**Table A2:** The baseline characteristics of the study population.

Age in Years	Distribution	Pregnancies				Miscarriages (in Total in the Age Group)	Births			
		1	2	3	≥4		1	2	3	≥4
18–24	3.7% (9 participants)	8	1	-	-	1	9	-	-	-
25–34	50.6% (122 participants)	67	39	13	3	27	93	26	3	-
35–44	29% (70 participants)	28	20	13	9	27	41	21	6	2
45–55	16.6 (40 participants)	10	18	8	4	14	12	23	4	1
