# Supplementary material for: Maternal Vaccination in Lithuania: A Cross-Sectional Study
Source: Vaccines (Basel). 2026 Apr 18;14(4):363. doi: 10.3390/vaccines14040363 (PMC13119657; doi:10.3390/vaccines14040363)
Supplement: Supplementary file 1 [file vaccines-14-00363-s001.zip › vaccines-4161209-supplementary.pdf]

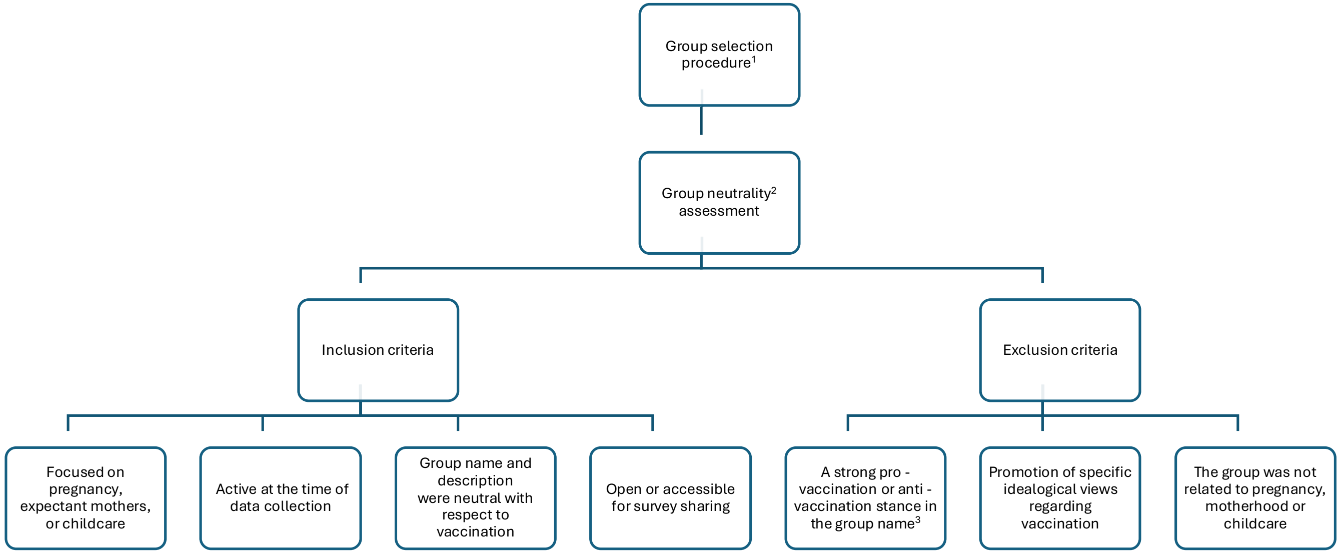

Figure S1: Selection of online groups for survey distribution

[1]Facebook groups and online forums were identified using keyword-based search in the Lithuanian language. The following keywords were used to identify relevant groups: nėštumas (pregnancy), nėščiosios (pregnant women), būsimos mamos (expectant mothers), mamų grupė (mothers'

group), birth cohort–based groups (e.g., „Rugsėjinukai 2025“, „Spalinukai 2025“), and vaikų forumai (child-related forums). Although predefined criteria were applied, the assessment of group neutrality may involve some degree of subjective judgment.

Only groups clearly related to pregnancy, motherhood, or childcare were considered eligible.

[2]Group neutrality was assessed based on the following: group name, group description (if available), initial review of publicly available posts and discussion topics. Groups were considered neutral if they did not promote a clear stance for or against vaccination and were primarily focused on general pregnancy, parenting, or childcare topics.

[3] Groups were excluded if the group name or description indicated a strong pro-vaccination or anti-vaccination stance, including keywords such as „vakcinų žala“ (vaccine harm) or „antivakseriai“ (antivaxxers), as well as explicitly pro-vaccination advocacy groups.

Table S1: Education, maternal age and vaccination rates.

**Education**	**Influenza vaccine administrated (n=241)**	**%***	**Tdap vaccine administrated (n=176)**	**%***	**RSV vaccine administrated (n=241)**	**%***
University degree (n=197)	59/197	30	67/139	48.2	7/197	3.6
Higher education (n=21)	5/21	23.8	5/18	27.8	1/21	4.8
High school education (n=21)	5/21	23.8	5/17	29.4	2/21	9.5
Compulsory education up to grade 10 (n=2)	0/2	0	0/2	0	0/2	0
P value	0.340	-	0.020	-	0.330	-
**Maternal age**						
18 – 24 (n=9)	3/9	33.3	4/7	57.1	2/9	22.2
25 – 34 (n=122)	44/122	36.1	50/105	0.5	6/122	4.9
35 – 44 (n=70)	21/70	30	23/53	43.4	1/70	1.4
45 – 55 (n=40)	1/40	2.5	0/11	0	1/40	2.5
P value	< 0.001	-	0.009	-	0.067	-

*Percentages represent the proportion of vaccinated individuals within each subgroup. P-values represent overall associations between education level or maternal age and vaccination uptake across categories.

Table S2: Reasons for not vaccinating.

Reasons for not vaccinating:	The amount of answers, %
There is insufficient data on vaccine safety.	19.2 %
I avoid any medications or vaccines whenever possible.	21.1 %
I am afraid of pain experienced during vaccination.	0.9 %
I am concerned about side effects caused by vaccines.	15 %
I am afraid of complications resulting from vaccination.	14.1 %
I am afraid that my child might contract the disease caused by the pathogen in the vaccine	3.2 %
I believe it is unlikely that my child will contract or suffer severely from the diseases for which vaccination is offered	5.6 %
I believe vaccination is ineffective	5.2 %
I am afraid that vaccines may weaken my child's immune system	5.6 %
I believe that children should acquire immunity naturally by contracting diseases	8 %
My family/friends/environment are skeptical about vaccination	1.4 %
The price for vaccination	0.5 %

Figure S2: Information sources influencing the decision to vaccinate during pregnancy.

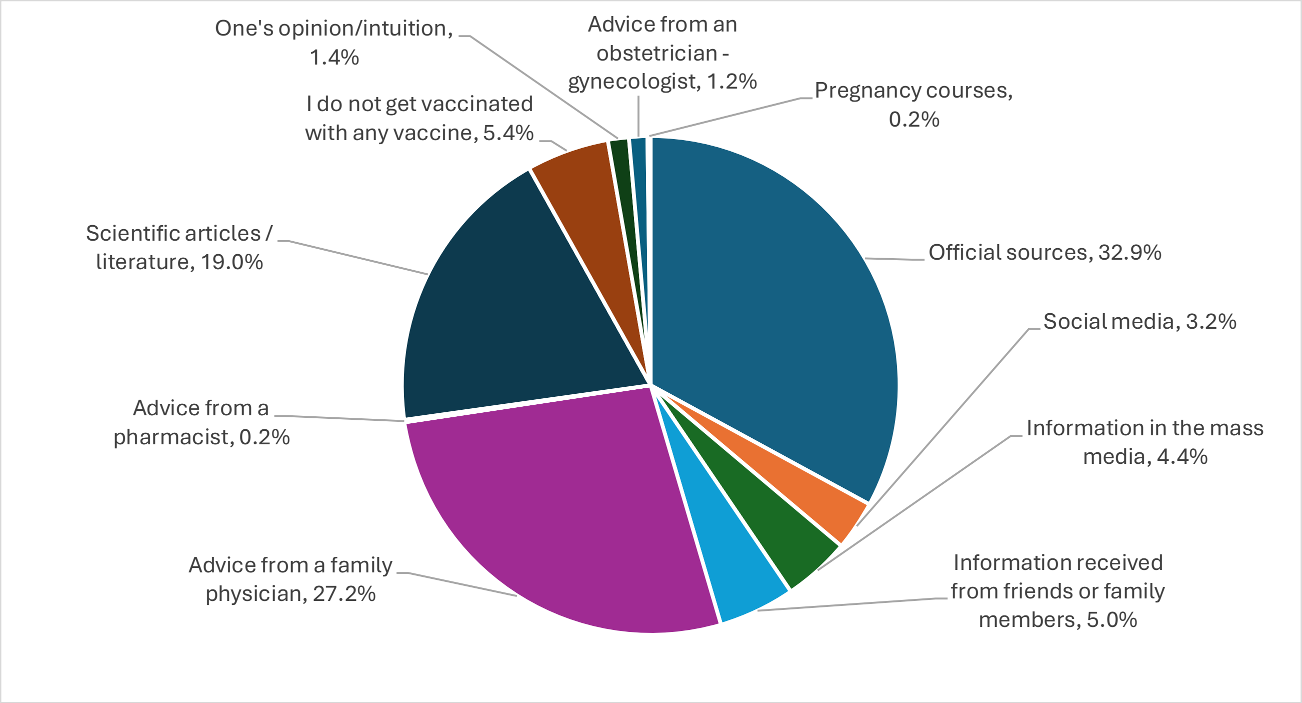

Figure S3: Information sources influencing the decision not to vaccinate during pregnancy.

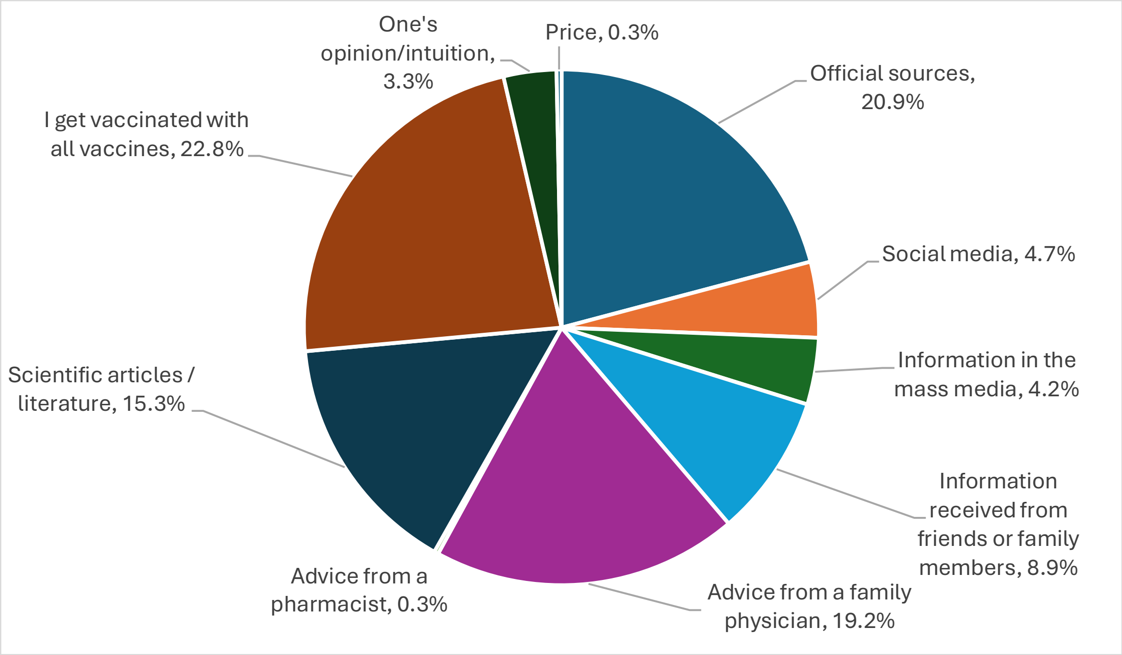

Figure S4: "Is it better to acquire immunity through natural infection or vaccination?'' Responses according to respondents

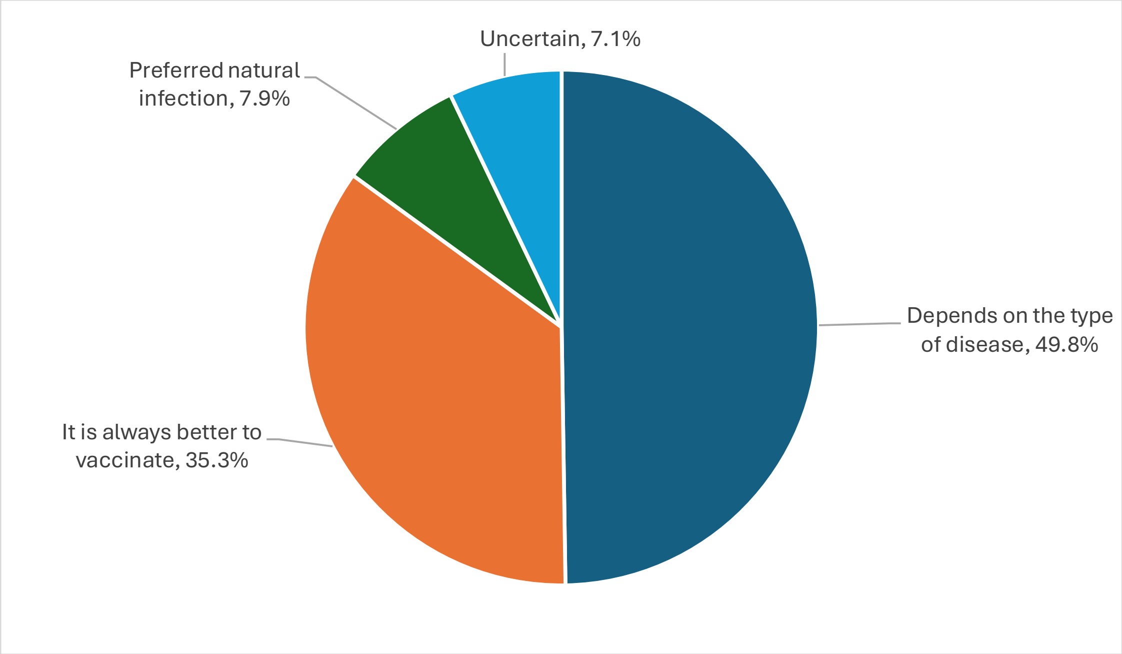

Table S3: ′′Should the vaccination for pregnant women be mandatory?′′ Responses according to respondents

Mandatory vaccination	Percentage of respondents, %
Agree	27.4 %
Disagree	48.5%
Unsure	24.1%

Table S4: Understanding the knowledge respondents of vaccine mechanisms

Vaccine can cause a disease	Percentage of respondents, %
In rare cases, live attenuated vaccines can cause disease	38.2%
Vaccines cannot cause disease at all	22.4%
Vaccines only mimic infection and therefore never cause the disease itself	19.5%
In rare cases, vaccines without live pathogens could cause disease	11.2%
Vaccines cause disease but protect from reinfection	8.7%

Table S5: ′′Vaccinations are beneficial because they protect against infectious diseases′′. Responses according to respondents

Vaccinations are beneficial because they protect against infectious diseases	Percentage of respondents, %
Agree	67.7%
Disagree	17.8%
Unsure	14.5%

Table S6: “Vaccines are safe”. Responses according to respondents

Vaccines are safe	Percentage of respondents, %
Agree	58.9%
Disagree	23.7%
Unsure	17.4%

Table S7: “Vaccines are only beneficial to pharmaceutical companies and not to vaccinated people”. Responses according to respondents.

Vaccines are only beneficial	Percentage of respondents, %
Agree	17%
Disagree	69.7%
Unsure	13.3%
